# A team effort: natural killer cells on the first leg of the tumor immunity relay race

**DOI:** 10.1186/s40425-018-0380-4

**Published:** 2018-07-09

**Authors:** Timothy B. Fessenden, Ellen Duong, Stefani Spranger

**Affiliations:** 1 Koch Institute for Integrative Cancer Research at MIT, 500 Main Street, Cambridge, MA 02139 USA; 2 Massachusetts Institute of Technology, Department of Biology, Cambridge, USA; 3 Howard S. and Linda B. Stern Career Development Professor, 77 Massachusetts Avenue, Cambridge, MA 02139 USA

## Abstract

Recent work by Böttcher and colleagues defines a new role for Natural Killer cells in the anti-tumor immune response, arriving early into the tumor microenvironment before passing the baton to DC1 dendritic cells. DC1 dendritic cells subsequently activate CD8+ T cells resulting in effective anti-tumor immunity. This work highlights the cooperative nature of anti-tumor immunity set in motion by Natural Killer cells, and immune evasion by tumors through their exclusion.

## Main text

Sustained infiltration of CD8+ T cells into the tumor microenvironment has been shown to generate an immune suppressive microenvironment through the upregulation of immune inhibitory molecules, including PD-L1 (programmed death receptor ligand 1) on tumor cells or myeloid cells [1]. Accordingly, the majority of melanoma patients with a T-cell inflamed tumor microenvironment responds to checkpoint blockade therapy targeting immune inhibitory pathways such as PD-1:PD-L1 interactions, while non-T cell-inflamed tumors tend to be refractory to this therapy [2]. Unraveling the cellular and molecular determinants of T cell recruitment to and retention in solid tumors is therefore a crucial step toward improving existing immunotherapies.

Antigen-presenting cells, in particular dendritic cells (DC), are known to orchestrate initial activation of T cells in the tumor-draining lymph node [1]. In particular, cross-presenting CD103+ DC (also known as DC1 or Clec9a + DC) appear to be critical for priming CD8+ T cells [3]. Recent reports have defined a distinct role for CD103+ DC residing within the tumor microenvironment, where they cooperate with CD8+ cytotoxic T cells to recognize and clear tumor cells. Consequently, tumors able to exclude CD103+ DC can successfully evade T-cell mediated immune control [4, 5]. However, our understanding of the recruitment and retention of tumor-resident DC populations is still sparse.

Böttcher et al. addressed this question using a melanoma tumor model previously reported to exclude T cells and CD103+ DC through the upregulation of cyclooxygenase enzymes COX-1 and COX-2 (encoded by *ptgs1* and *ptgs2*) and their catalytic product, prostaglandin E2 (PGE2) [6]. In addition to the exclusion of CD103+ DC, they observed a stark reduction in the recruitment of Natural Killer (NK) cells into tumors [7]. Assessing the chemokine expression profile of tumor-residing immune cell subsets, the authors showed that XCL1 is produced exclusively by NK cells while CCL5 is secreted by both NK cells and CD8+ effector T cells. Both XCL1 and CCL5 as well as CCL4 have previously been reported to attract CD103+ DC to inflammatory sites through binding to XCR1/CCR5 chemokine receptors [4, 8], results that were confirmed in this study. In contrast to observations by Böttcher et al., however, previous studies found chemokine production originated from non-immune cells, suggesting that the source for chemokines might be highly context dependent.

Depletion of NK cells resulted in failed recruitment of CD103+ DC and immune evasion by tumors. In vitro analysis showed that tumor-derived PGE2 resulted in diminished secretion of CCL5 and XCL1 by NK cells and increased NK cell death [7]. The observation of increased lymphocyte death in tumors with high levels of PGE2 agrees with previous reports. A study of ovarian cancer found that COX-2/PGE2 signaling caused increased expression of Fas-ligand on endothelial cells, which in turn increased apoptosis of infiltrating T cells and resulted in a non-T cell-inflamed tumor microenvironment [9]. Both studies show a cell death-related phenotype, however since NK cell death was observed ex vivo*,* the molecular mechanism appear to be distinct and should be fully elucidated.

The authors then validated their findings using patient samples and found that indeed NK cells are the dominant source of XCL1/2, while CD8+ effector T cells produce high levels of CCL5. Using human RNA transcript data, the authors provide further evidence supporting the concept that NK cells recruit CD103+ DC, which in turn are required for the recruitment of effector T cells. Previous work has shown that CD103+ DC are critical for the recruitment of effector T cells into the tumor microenvironment through the secretion of CXCL9 and CXCL10 [5, 6]. Likewise, these studies provided evidence that restoration of CD103+ DC infiltration mediates regained responsiveness to immunotherapy.

**Fig. 1:**
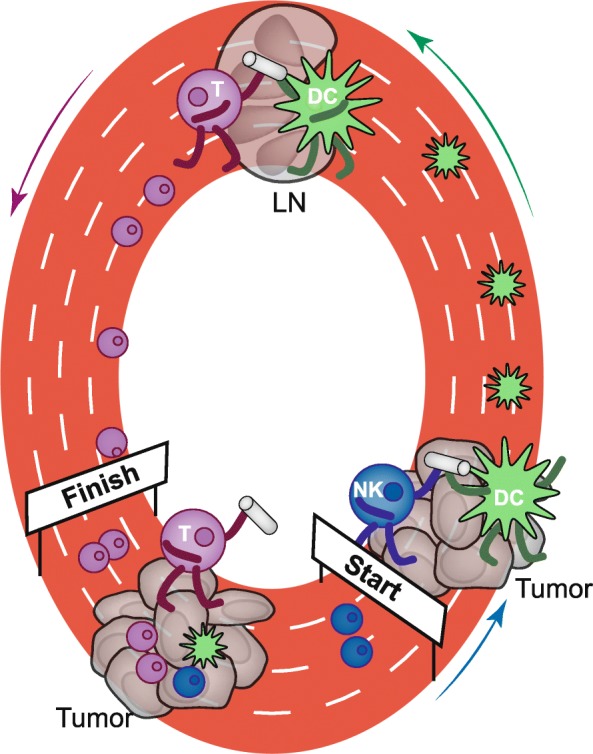
The tumor-immune relay race. Natural Killer (NK) cells are the first to arrive in the tumor microenvironment and recruit CD103+ dendritic cells (DC) through the secretion of chemokines. Activated DC then transport antigen from the tumor to the tumor-draining lymph node where they prime T cells (T). DC residence in tumors is also critical to drive effector T cell recruitment into the tumor microenvironment. Sensing and eradication of tumors is thus the result of collaboration of distinct cell types within the tumor microenvironment

Considering these observations in conjunction with those made by Böttcher and colleagues, it is plausible to suggest that anti-tumor immunity may operate as a relay race in which immune cells recruit each other to pass the tumor-reactive “baton” (See Fig. 1). Such cooperation ensures the sequential recruitment of NK cells, CD103+ DC, and most importantly CD8+ effector T cells into the tumor microenvironment. It is worth noting that, similar to effector T cells, NK cells can also be recruited by CXCL9 and CXCL10.

Meanwhile, CD8+ effector T cells are also a source of CCL4, CCL5 and XCL1. This redundancy in chemokine-mediated immune cell recruitment suggests a strong positive feedback loop, and highlights the importance of the recruitment and activation of early effector cells such as NK cells.

The canonical cytolytic role of NK cells can directly or indirectly contribute to tumor control.

However, Böttcher et al. make several intriguing observations regarding the role of NK cells during an anti-tumor immune response that go beyond their classically described function. The intriguing notion that innate lymphocytes can be recruited into the tumor at very early stages could explain the recruitment of CD103+ DC into tumors lacking chemokine production. In conjunction with the observation that PGE2 inhibits NK cell function, it could therefore be speculated that PGE2 mediates inhibition of anti-tumor immune responses at early stages of tumor development. Indeed, expression of cyclooxygenases and production of PGE2 has been linked to malignant transformation in multiple tissues and therefore is an early event in tumor development [10]. However, additional studies will be needed to elucidate how NK cells are activated and recruited into early tumor lesions. Similarly, whether NK cell activation is required in all cancer types to drive recruitment and retention of CD103+ DC and CD8+ effector T cells merits further investigation. Determining the mechanism of NK cell recruitment could potentially lead to direct targeting of NK cell recruitment as a therapeutic target to start the tumor-immune “relay race.” This therapeutic approach could convert immunotherapy-resistant, non-T cell-inflamed tumors into checkpoint blockade therapy-sensitive T cell-inflamed microenvironments, potentially independent of the tumor type and molecular mechanism for exclusion of CD103+ DC and CD8+ T cells.
